# Targeted therapies in genetic dilated and hypertrophic cardiomyopathies: from molecular mechanisms to therapeutic targets. A position paper from the Heart Failure Association (HFA) and the Working Group on Myocardial Function of the European Society of Cardiology (ESC)

**DOI:** 10.1002/ejhf.2414

**Published:** 2022-01-14

**Authors:** Rudolf A. de Boer, Stephane Heymans, Johannes Backs, Lucie Carrier, Andrew J.S. Coats, Stefanie Dimmeler, Thomas Eschenhagen, Gerasimos Filippatos, Lior Gepstein, Jean‐Sebastien Hulot, Ralph Knöll, Christian Kupatt, Wolfgang A. Linke, Christine E. Seidman, C. Gabriele Tocchetti, Jolanda van der Velden, Roddy Walsh, Petar M. Seferovic, Thomas Thum

**Affiliations:** ^1^ Department of Cardiology University Medical Center Groningen, University of Groningen Groningen The Netherlands; ^2^ Department of Cardiology Maastricht University Medical Center (MUMC+) Maastricht The Netherlands; ^3^ Department of Cardiovascular Sciences University of Leuven Leuven Belgium; ^4^ Institute of Experimental Cardiology Heidelberg University Heidelberg Germany; ^5^ German Center for Cardiovascular Research (DZHK), Partner Site Heidelberg/Mannheim Heidelberg Germany; ^6^ Department of Experimental Pharmacology and Toxicology University Medical Center Hamburg‐Eppendorf Hamburg Germany; ^7^ German Centre for Cardiovascular Research (DZHK), partner site Hamburg/Kiel/Lübeck Hamburg Germany; ^8^ Faculty of Medicine University of Warwick Warwick UK; ^9^ Institute for Cardiovascular Regeneration Goethe University Frankfurt Germany; ^10^ German Center for Cardiovascular Research (DZHK) Frankfurt Germany; ^11^ Cardio‐Pulmonary Institute (CPI) Frankfurt Germany; ^12^ Department of Cardiology, National and Kapodistrian University of Athens, School of Medicine Attikon University Hospital Athens Greece; ^13^ Department of Cardiology, Rambam Health Care Campus Technion ‐ Israel Institute of Technology Haifa Israel; ^14^ Université de Paris, INSERM, PARCC Paris France; ^15^ CIC1418 and DMU CARTE, AP‐HP Hôpital Européen Georges‐Pompidou Paris France; ^16^ Department of Medicine, Integrated Cardio Metabolic Centre (ICMC), Heart and Vascular Theme Karolinska Institute Stockholm Sweden; ^17^ Bioscience, Cardiovascular, Renal & Metabolism, BioPharmaceuticals R&D, AstraZeneca Gothenburg Sweden; ^18^ Department of Cardiology University Clinic rechts der Isar, Technical University of Munich, Germany and German Center for Cardiovascular Research (DZHK), Munich Heart Alliance Munich Germany; ^19^ Institute of Physiology II University Hospital Muenster Muenster Germany; ^20^ Department of Genetics Harvard Medical School Boston MA USA; ^21^ Cardiovascular Division, Department of Medicine Brigham and Women's Hospital Boston MA USA; ^22^ Howard Hughes Medical Institute Harvard University Boston MA USA; ^23^ Department of Translational Medical Sciences, Center for Basic and Clinical Immunology Research (CISI); Interdepartmental Center for Clinical and Translational Research (CIRCET); Interdepartmental Hypertension Research Center (CIRIAPA) Federico II University Naples Italy; ^24^ Department of Physiology, Amsterdam UMC, Amsterdam Cardiovascular Sciences Vrije Universiteit Amsterdam Amsterdam The Netherlands; ^25^ Department of Clinical and Experimental Cardiology, Amsterdam UMC, Amsterdam Cardiovascular Sciences University of Amsterdam, Heart Center Amsterdam The Netherlands; ^26^ Serbian Academy of Sciences and Arts Belgrade Serbia; ^27^ Faculty of Medicine University of Belgrade Belgrade Serbia; ^28^ Institute of Molecular and Translational Therapeutic Strategies Hannover Medical School Hannover Germany; ^29^ Fraunhofer Institute for Toxicology and Experimental Medicine Hannover Germany

**Keywords:** Cardiomyopathy, Dilated cardiomyopathy, Hypertrophic cardiomyopathy, Disease mechanism, Pharmacology, Gene therapy, Molecular biology, Heart failure

## Abstract

Genetic cardiomyopathies are disorders of the cardiac muscle, most often explained by pathogenic mutations in genes encoding sarcomere, cytoskeleton, or ion channel proteins. Clinical phenotypes such as heart failure and arrhythmia are classically treated with generic drugs, but aetiology‐specific and targeted treatments are lacking. As a result, cardiomyopathies still present a major burden to society, and affect many young and older patients. The Translational Committee of the Heart Failure Association (HFA) and the Working Group of Myocardial Function of the European Society of Cardiology (ESC) organized a workshop to discuss recent advances in molecular and physiological studies of various forms of cardiomyopathies. The study of cardiomyopathies has intensified after several new study setups became available, such as induced pluripotent stem cells, three‐dimensional printing of cells, use of scaffolds and engineered heart tissue, with convincing human validation studies. Furthermore, our knowledge on the consequences of mutated proteins has deepened, with relevance for cellular homeostasis, protein quality control and toxicity, often specific to particular cardiomyopathies, with precise effects explaining the aberrations. This has opened up new avenues to treat cardiomyopathies, using contemporary techniques from the molecular toolbox, such as gene editing and repair using CRISPR‐Cas9 techniques, antisense therapies, novel designer drugs, and RNA therapies. In this article, we discuss the connection between biology and diverse clinical presentation, as well as promising new medications and therapeutic avenues, which may be instrumental to come to precision medicine of genetic cardiomyopathies.

## Introduction

Cardiomyopathies form a heterogeneous group of heart muscle diseases and an important cause of heart failure (HF). Comprehensive understanding of the underlying mechanisms and natural courses have led to recent therapeutic advances with far‐reaching impact on management and prognosis.^1^

Amongst the cardiomyopathies, hypertrophic and dilated cardiomyopathy (HCM, DCM, respectively) are the most prevalent. Recently, the epidemiology of genetic cardiomyopathies has extensively been reviewed and summarized.^2^, ^3^

The prevalence of HCM is 2–5 per 1000 of the general population.^4^, ^5^, ^6^, ^7^ In 40%–50% of patients, HCM results from autosomal dominant sarcomere gene mutations, whereas other aetiologies, including hereditary syndromes, neuromuscular disorders and storage diseases, which are considered as phenocopies of HCM, account for 5%–10% of patients.^3^, ^8^, ^9^ Most patients with HCM have an asymmetric septal hypertrophy, and approximately 40%–70% have an obstructive HCM (at rest or during exercise), and significant left ventricular (LV) intracavitary reduction.^10^, ^11^ Non‐obstructive HCM is present in 30%–60% patients. Timely recognition and treatment of patients at risk for sudden cardiac death or progressive HF is important. Recently, the EXPLORER‐HCM trial introduced mavacamten, a selective allosteric inhibitor of cardiac myosin ATPase, which successfully reduced outflow obstruction of HCM.^12^

Dilated cardiomyopathy primarily presents as HF with reduced ejection fraction (HFrEF), and advanced HF in DCM is one of the leading indications for heart transplantation. The prevalence of DCM in Europe and North America has initially been underestimated, at numbers of 6–15/100 000.^13^, ^14^ However, we have dramatically improved our use of sensitive imaging modalities and there has been a revolution of next genome sequencing with better yield of genetic testing. A more general observation is that DCM is approximately twice as prevalent as HCM. So, likely, the true prevalence is between 1:2500 at the minimum and 1:250 at the maximum.^13^, ^15^ The pathophysiology of DCM includes genetic causes, as well as direct myocardial damage caused by infectious or toxic agents, endocrine and metabolic abnormalities, immune‐mediated processes and peripartum cardiomyopathy (PPCM). Newer treatment options, on top of guideline‐directed medical therapy (GDMT), include bromocriptine in PPCM while treatments targeted at the immune response and autoantibodies are still under investigation.^16^

The prevalence of restrictive cardiomyopathy (RCM) is currently unknown, but it is the least frequent amongst the cardiomyopathies.^17^ The aetiology of RCM is heterogeneous, including idiopathic, hereditary and acquired cases of non‐infiltrative and infiltrative myocardial disorders, storage diseases and endomyocardial disorders. HF occurs frequently (as high as 83%) in those patients,^18^ and the majority of patients present with HF with preserved ejection fraction (HFpEF), while HFrEF usually occurs at a later stage (e.g. in amyloidosis or iron overload/haemochromatosis). Recently, new treatment insights with novel aetiology‐specific therapies were introduced, including transthyretin stabilizers in cardiac amyloidosis^19^, ^20^ and enzyme replacement therapies (Anderson–Fabry^21^ and Pompe disease^22^).

In this review, we have focused on genetic HCM and DCM, and specifically highlighted genetic mutations in which strong scientific interest exist, with recent progress, resulting in potential targeted therapeutic modalities, either in development or entering the clinical arena. We clearly acknowledge that many more (rare) forms of cardiomyopathies exist and are subject to intensive study, with new treatments on the horizon. For some, e.g. myocardial amyloid disease, there is a very wide body of literature and recent reviews have covered this topic extensively,^23^, ^24^, ^25^ and given space restraints, we refer to this work. For other (very rare) diseases, such as RCM and arrhythmogenic right ventricular cardiomyopathies, recent progress is limited.^26^, ^27^, ^28^, ^29^ Causes of RCM include genetic HCM, cardiac amyloidosis, chemotherapy or chest exposure to radiotherapy, cardiac haemochromatosis, and rarely cardiac sarcoidosis. The therapy as such will depend of the underlying cause, considering tafamidis or RNA silencing therapy in transthyretin amyloidosis and treating haemochromatosis and sarcoidosis lege artis.^26^ Left ventricular non‐compaction (LVNC), a classified form of cardiomyopathy, is a genetic disease characterized by excessive and unusual trabeculations within the mature left ventricle.

Left ventricular non‐compaction is quite commonly found in families where other affected relatives have typical HCM or DCM. Therefore, LVNC is seen as a phenotypic variation of genetic variants otherwise related to the DCM/HCM phenotype. Shared molecular mechanisms or treatment of LVNC will likely have an overlap with HCM and DCM.

**Figure 1:**
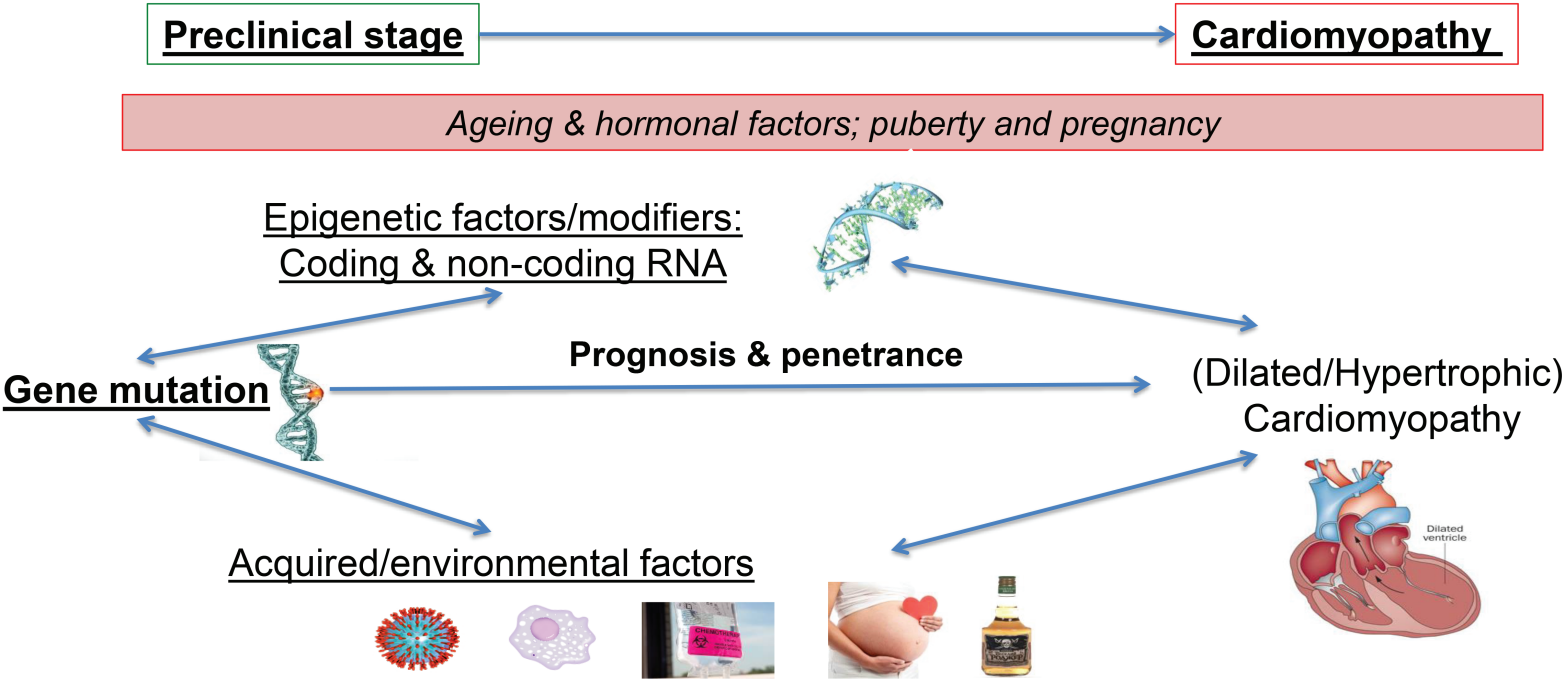
The multiple hit model of cardiomyopathy phenotype assumes that the phenotype is explained by the genetic mutation underpinning the disorder, and an additional ‘second hit’ by an environmental or genetic factor. These ancillary (epi‐)genetic or environmental factors may accelerate or attenuate the phenotype.

Although in many cardiomyopathies a pathogenic genetic variant is the main trigger, there is remarkable variance between individuals, with differences in age‐dependent penetrance, and it is believed that ancillary (genetic, environmental) triggers are needed to provoke a full‐blown phenotype (second hit hypothesis) (*Figure* *1*). In the clinical work‐up, an important interaction between genetic and acquired causes needs to be considered. Identification of an acquired cause of the cardiomyopathy does not exclude an underlying pathogenic gene variant, whereas the latter may require an additional acquired cause and/or disease modifier to become manifested clinically.^30^

While insights in the exact pathomechanisms of cardiomyopathies have increased, the treatment is still predominantly following a one‐size‐fits‐all regimen, with non‐specific drugs such as beta‐blockers being widely employed. From a pathophysiological point of view, it is remarkable that a large majority of the patients do respond to generic drug regimen. However, many patients do not adequately respond, and specific sub‐forms are not or marginally amenable to standard therapies, and would benefit from targeted treatments. The Translational Committee of the Heart Failure Association (HFA) of the European Society of Cardiology (ESC) organized a workshop to discuss the most recent insights and to foster a greater awareness of these conditions, given the high potential of new targeted drugs on the horizon.

## Opportunities to intervene – new insights from specific disease features

### Monogenic drivers of dilated cardiomyopathy with specific pathomechanisms

#### Phospholamban cardiomyopathy: a ‘cardiodegenerative disease’ of protein aggregation

Phospholamban (PLN) is a protein in the sarcoplasmic reticulum membrane that regulates the sarco−/endoplasmic reticulum Ca^2+^‐ATPase (SERCA) protein. PLN plays crucial roles in calcium handling in health and disease.^31^, ^32^ A specific mutation in the *PLN* gene, where the arginine in amino acid position 14 of the PLN protein is deleted (p.Arg14del), results in a gain of function of PLN with chronic suppression of SERCA activity. The consequent cardiomyopathy is common in the Netherlands and the United States, and gives rise to a severe form of cardiomyopathy with high risk of developing arrhythmias and end‐stage HF.^33^, ^34^ Of individuals with the PLN p.Arg14del variant, between 20%–40% will develop life‐threatening ventricular arrhythmia or HF (of whom approximately 15% dies) within 5 years. The exact rate will depend on the extent of family screening, with identification of phenotype‐negative mutation carriers. Patients with a PLN mutation have a higher frequency of LV structural and functional abnormalities compared to other genetic arrhythmogenic cardiomyopathy patients.^35^ Decreased LV ejection fraction (LVEF), the number of premature ventricular contractions and presence of negative T waves on the electrocardiogram (ECG) are the main predictors of malignant ventricular arrhythmias and death.^36^ So the reported rate of cardiac mortality will depend on the functional/electric penetrance of the mutation along the extent of family screening (asymptomatic vs. symptomatic; phenotype negative vs. positive).

Phospholamban cardiomyopathy is characterized by common features of severe HF, such as cell loss, fibrosis and impaired calcium handling. However, besides these common pathways, patients with PLN cardiomyopathy have typical PLN aggregates that localize with the endoplasmic reticulum and nucleus.^37^ A mouse model with the murine variant of the p.Arg14del mutation almost completely mimics the human cardiomyopathy.^38^ Studying these mice, PLN aggregates may occur already in the disease process, before an overt cardiac phenotype has ensued.^39^, ^40^ This ‘cardiodegenerative’ phenotype prompts further study, and may provide novel targets for disease monitoring and therapy. Currently, a large number of pre‐clinical studies are conducted, with compounds that aim to repair the genetic defect (e.g. antisense and gene therapy), but also that target protein aggregation and integrity, as these appear so crucial to PLN cardiomyopathy.^41^, ^42^

#### Laminopathies – targeting the core of the cardiomyocyte?

Mutations in the lamin A/C (*LMNA*) gene cause laminopathies, a group of disorders characterized by phenotypically heterogeneous manifestations. Laminopathies either specifically affect distinct tissues (striated muscles, peripheral nerves, the adipose tissue), or present as a systemic disease (progeria, or accelerated ageing) involving several organs. Cardiac involvement is one of the most prevalent and severe manifestations, with a rapidly progressing DCM associated with conduction system disease, atrial fibrillation (AF) and malignant ventricular tachyarrhythmias.^43^ In DCM patients with pathogenic LMNA variants, between 15%–40% will develop life‐threatening ventricular arrhythmias (approximately 15% dies) or HF progression within 5 years. As for PLN, also non‐sustained ventricular tachycardia, and a decreased LVEF, along male sex and non‐missense mutations, are the main risk factors.^36^

Lamin A/C is a nuclear protein, and mutations cause structural abnormalities in the nuclear envelope, triggering different molecular events underlying LMNA‐related DCM.^44^ The latter includes activation of the MAP kinases (Ras, ERK1/2, p38 MAPK) and AKT/mTOR, chromatin disorganization and increased platelet‐derived growth factor receptor (PDGFR) expression, all causing myocyte death and fibrosis.^45^ Current clinical trials have focused on the inhibition of p38 MAPK, Ras, mTOR and PDGFR, with an ongoing phase 3 trial involving a selective oral inhibitor of the p38 MAPK pathway in symptomatic LMNA‐DCM (ARRY‐371797, also PF‐07265803; https://clinicaltrials.gov/ct2/show/NCT03439514). Recently, a Ras‐inhibitor (lonafarnib) – Ras/MAPK being crucial in signal transduction of cell growth and differentiation – has been approved to reduce mortality in LMNA‐related progeria (Hutchinson–Gilford progeria syndrome and progeroid laminopathies).^46^ Whether this would also be of benefit in LMNA‐DCM has not yet been studied. Given the pleiotropic effects of mutations in the LMNA gene, initiatives have been launched to bundle knowledge on new therapies, and a substantial number of novel treatment modalities has been described, although most trials and case series are limited in patient numbers.^47^ As an example of this it was shown that LMNA mutation‐associated endothelial dysfunction could be improved by lovastatin treatment.^48^

#### Titin truncation‐cardiomyopathy: do mechanisms guide future clinical trials?

**Figure 2:**
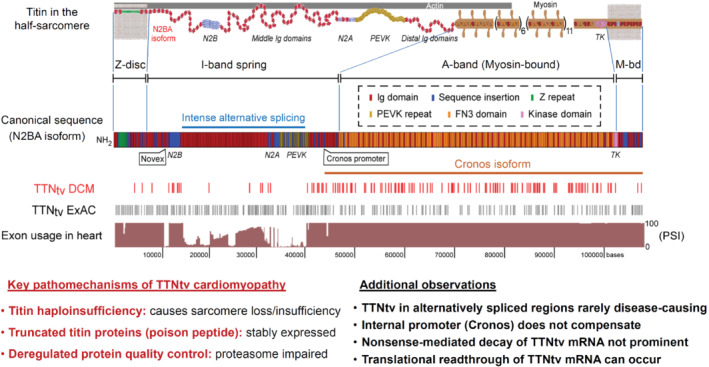
Current paradigm of titin protein homeostasis, in particular for truncating variants (TTNtv). This involves haploinsufficiency, truncated titin proteins, and impaired protein quality control.^63^ Usage of *TTN* exons in human heart and location of *TTN* truncations detected in dilated cardiomyopathy (DCM) patient or control cohorts (hatch marks) shown according to Schafer et al.^54^ Modified from Linke.^49^

Titin (*TTN*) is one of the largest proteins encoded by the human genome, and is essential for cardiomyocyte structure and function.^49^ In 15%–25% of patients with DCM, heterozygous *TTN*‐truncating variants (TTNtv) underlie the disease,^50^, ^51^, ^52^ and at least half of these variants are localised in non‐transcribed exons.^53^ However, TTNtv are also present at a rate of 0.5%–3% in the healthy population, where they pose a risk factor for adverse cardiac remodelling.^54^ Thus, a TTNtv may be more pathogenic if additional genetic or acquired/environmental factors contribute to disease development.^55^ In fact, TTNtv are present in up to 15% of patients with ‘acquired DCM’, including pregnancy‐,^52^ alcohol‐^56^ and chemotherapy‐induced^57^ cardiomyopathy (*Figure* *1*). TTNtv also predispose to early or familial AF^58^ and ventricular tachyarrhythmias.^59^, ^60^ TTNtv can as such be regarded as a common genetic risk factor to develop HF and/or arrhythmias when combined with additional acquired (or genetic) disease factors (*Figure* *2*).

Importantly, cardiac function improves in up to 80% of TTNtv cardiomyopathy patients upon optimal HF treatment, but the other 20% still follows a more malignant clinical course, with persistent cardiac dysfunction and life‐threatening arrhythmias.^61^ RNA sequencing (RNA‐seq) on right septal biopsies gathered during diagnostic work‐up in DCM patients carrying a pathogenic genetic variant (*TTN*, *LMNA*, *RBM20* or *MYH7*) have revealed clearly distinct transcriptome profiles in TTNtv and *LMNA*‐DCM, as shown by a principal component analysis. In addition, two distinct subgroups are present within the TTNtv patients. The transcriptomic profile of the malignant TTNtv group (lower LVEF, increased cardiac fibrosis, more arrhythmias, a higher concentration of blood inflammation markers, and N‐terminal pro‐B‐type natriuretic peptide) clustered close to LMNA patients, with similarities in their clinical phenotype.^62^

Recent work has elucidated the key pathomechanisms of TTNtv‐DCM: protein studies in over 100 end‐stage failing DCM heart tissues, 22 of them with a TTNtv, suggested that titin haploinsufficiency is present in TTNtv hearts and causes loss of sarcomeres (*Figure* *2*).^63^, ^64^ Moreover, truncated titin proteins were shown to be stably expressed in adult TTNtv patient heart tissue, and their content was higher in more severe disease.^63^ Thus, a dominant‐negative ‘poison peptide’ effect is likely, with associated deregulated protein quality control, notably proteasomal dysfunction and aggregate formation. Contractile deficiency was shown to be present in human induced pluripotent stem cell‐derived cardiomyocytes (hiPSC‐CMs) with a heterozygous TTNtv engineered into three‐dimensional heart muscle.^63^, ^64^ Repair of the TTNtv by CRISPR/Cas9 gene editing in engineered heart muscle fully rescued contractility.^63^

#### The case for ‘phenomapping’

This heterogeneity in DCM phenotypes as a consequence of a mix of genetic and acquired factors, makes it difficult to classify DCM with great precision, and hence to guide clinical decision‐making. Therefore, Vendonschot and colleagues recently included 795 consecutive DCM patients from the Maastricht Cardiomyopathy Registry who underwent in‐depth phenotyping, comprising extensive clinical data on aetiology and comorbidities, imaging, genetics and endomyocardial biopsies for unsupervised machine learning clustering.^65^ Four mutually exclusive and clinically distinct phenogroups (PG) were identified based upon unsupervised hierarchical clustering of principal components: [PG1] mild systolic dysfunction, [PG2] auto‐immune, [PG3] genetic and arrhythmias, and [PG4] severe systolic dysfunction. RNA‐seq of cardiac samples revealed a distinct underlying molecular profile per PG: pro‐inflammatory (PG2, auto‐immune), pro‐fibrotic (PG3; arrhythmia‐genetic) and metabolic (PG4, low ejection fraction) gene expression. Identification of DCM phenogroups associated with significant differences in clinical presentation, underlying molecular profiles, and outcome, will help to pave the way for individual treatment. In particular, genetic modifiers may be discovered by large genome‐wide association studies (GWAS), and these have already generated several genetic loci associated with early onset DCM.^66^, ^67^ Still, studies to determine the additional genetics (epigenetic and transcriptional) in different cells (single cell/nuclear sequencing) along epidemiological data (acquired factors, family members) are required to come to individualized therapy for DCM patients.

## Monogenic drivers of hypertrophic cardiomyopathy with specific pathomechanisms

**Figure 3:**
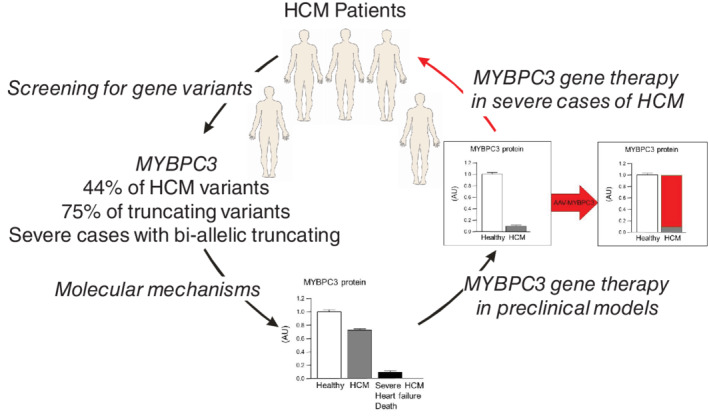
From bedside to bench and back to the patients for severe forms of hypertrophic cardiomyopathy (HCM) with *MYBPC3* truncating mutations.

Gene mutations encoding cardiac contractile proteins account for 30%–60% of the aetiology of HCM, characterized by LV hypertrophy, increased interstitial fibrosis and diastolic dysfunction.^68^ *MYBPC3* and *MYH7*, encoding cardiac myosin‐binding protein C (cMyBP‐C) and β‐myosin heavy chain, are the most prevalently affected genes, containing approximately 30%–40% of all pathogenic or likely pathogenic HCM variants, respectively.^69^ *TNNT2*, which encodes cardiac troponin T, is the main affected thin filament gene. Most of the *MYBPC3* mutations are truncating (75%),^70^, ^71^, ^72^ and their expression is regulated at the mRNA level by non‐sense‐mediated decay (NMD),^73^ leading to the absence of mutant protein and cMyBP‐C haploinsufficiency.^74^, ^75^, ^76^ Individuals with bi‐allelic *MYBPC3* mutations develop a more severe form of HCM.^72^ In the case of bi‐allelic truncating mutations, the regulation of the expression of non‐sense mRNAs by NMD is expected to lead to complete deficiency of cMyBP‐C in the sarcomere. In patients, this results in a massive hypertrophy, severe systolic and diastolic dysfunction, progressive HF and death within 1 year. For these infants, there is no other therapy than heart transplant. Pre‐clinical data of *MYBPC3* gene therapy in HCM mouse models, mouse and human engineered heart tissues and hiPSC‐CMs illustrate that *MYBPC3* gene replacement therapy via adeno‐associated virus (AAV) vector transfer is appropriate to replace the deficient protein in cardiomyocytes for severe forms of HCM (*Figure* *3*).^77^, ^78^, ^79^, ^80^, ^81^, ^82^

In past years, several mutation‐mediated pathomechanisms have been deciphered that drive HCM. The mutation‐induced changes relate to a disturbance of cellular homeostasis at three different levels, protein, functional and structural, which may all be target for therapies. Evidence for perturbed regulation of protein homeostasis comes from studies in mouse and human. Both the ubiquitin‐proteasome system and autophagy‐lysosomal pathway are impaired in mouse models of HCM, and activation of autophagy partially restored the phenotype.^83^, ^84^ The mutation‐related changes in sarcomere function funnel to a disturbance of energy homeostasis. High calcium‐sensitivity, perturbed length‐dependent activation, increased cross‐bridge kinetics and increased tension cost of myofilament contraction^85^ may all increase ATP utilization by sarcomeres. The ATPase activity of the myosin heads represents the energy‐consuming process at the level of the sarcomeres. About 10 years ago, a new state of myosin was described, which was termed the super‐relaxed state (SRX), in skeletal muscle and subsequently in cardiac muscle.^86^, ^87^ This SRX state has a very low metabolic rate, and it was suggested that the SRX state could be a cardioprotective mechanism by decreasing the metabolic load on the heart. Subsequent studies in mouse and human showed a reduction of SRX in tissue with reduced cMyBP‐C,^88^, ^89^, ^90^ indicating that truncating *MYBPC3* mutations reduce SRX and thereby result in an energetically unfavourable myosin state. A shift from SRX to a disordered state (DRX) was recently also described for *MYH7* mutations.^91^ Overall, mutation‐induced sarcomere changes have a negative impact on cellular energetics and may be targeted by interventions aimed to correct the sarcomere deficit, or by optimizing processes involved in metabolism and mitochondrial function.^92^ Recent findings point to a marked accumulation of the microtubule network,^93^ an inverse correlation between the levels of cMyBP‐C and tubulin,^94^ and higher detyrosinated tubulin level in HCM.^95^, ^96^ A disturbance of the microtubule network, which has a central role in structure, function and transport in cardiomyocytes, represents a third mechanism with likely major impact on cardiomyocyte homeostasis in HCM.

**Figure 4:**
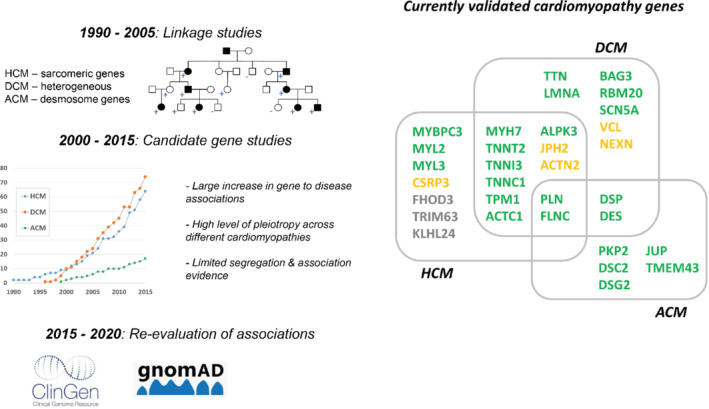
Summary of approaches used to define gene to disease associations for cardiomyopathies (*left*) and list of currently validated genes cardiomyopathies (*right*). Gene lists are based on ClinGen curation results, showing genes with definitive/strong evidence (green) or moderate evidence (orange). Genes in grey have been associated with hypertrophic cardiomyopathy (HCM) since the HCM ClinGen paper was published. For overlapping genes, the highest evidence class obtained is shown. ACM, arrhythmogenic cardiomyopathy; DCM, dilated cardiomyopathy.

The genes described above constitute some of the most prevalent and deeply characterized monogenic drivers of DCM and HCM. Dozens of additional genes have been implicated in cardiomyopathies in recent years, though these reports varied considerably in their study design and strength of evidence (the findings of candidate gene studies in particular often failed to replicate). Comprehensive reappraisal of these associations has been performed in recent years, through rare variant association studies^97^, ^98^ and curation of published evidence though the NIH‐funded ClinGen initiative.^99^, ^100^ The validated gene‐disease associations arising from these efforts reveal different genetic architectures that better reflect the distinctive phenotypes of the major cardiomyopathies, and enable more focused and accurate genetic testing for cardiomyopathy patients (*Figure* *4*).

The incomplete penetrance and variable expressivity associated with pathogenic cardiomyopathy variants, and the high proportion of cases that lack known pathogenic variants, suggest these conditions have a more complex genetic aetiology than allowed for by simple Mendelian inheritance. Two recent GWAS have revealed a key role for common genetic variation in HCM.^101^, ^102^ Estimates of heritability revealed a strong polygenic influence for sarcomere‐negative HCM (h^2^_g_ = 0.34 ± 0.02), and polygenic risk scores explained a significant portion of the phenotypic variability observed in cases with rare pathogenic variants. Intriguingly, these studies also demonstrated strong genetic correlation between HCM and DCM (as well as associated LV traits), but with opposite directions of effect – risk alleles for HCM were protective for DCM and vice versa. The shared genetic pathways for HCM and DCM revealed by these GWAS may identify novel targets for future therapeutic strategies.

## Opportunities to intervene – a new toolbox

### Human engineered heart tissues and induced pluripotent stem cells: improving our models

**Figure 5:**
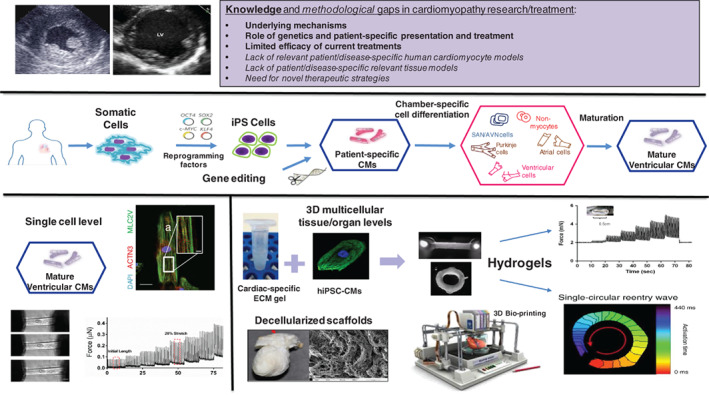
Toolbox in the study of genetic cardiomyopathies: from models on single cell level, to inducible pluripotent stem (iPS) cell modelling, to three‐dimensional and engineered heart tissue. CM, cardiomyocyte; hiPSC‐CM, human induced pluripotent stem cell‐derived cardiomyocyte.

The study of inherited cardiomyopathies has been hampered by the lack of relevant *in vitro* human cardiac cell or tissue models, particularly those reflecting patient‐specific abnormalities, by limitations in determining the role of genetic changes in disease pathogenesis, and by paucity of specific and effective therapies (*Figure* *5*). These challenges may be overcome through generation of patient‐specific hiPSC‐CMs.^103^ In the field of inherited cardiomyopathies, patient‐ or disease‐specific hiPSC‐CM models were established focusing on mutations in genes encoding cardiac sarcomeric and cytoskeletal proteins, ion channels, nuclear proteins, mitochondrial proteins and lysosomal proteins.^104^ Patient‐derived hiPSC‐CMs were able to recapitulate the different disease phenotypes in the culture‐dish, to provide mechanistic insights into disease processes, and to screen for existing and novel therapeutic measures. Combing the hiPSC‐CM technology with genome‐editing approaches (CRISPR/Cas system, see infra) provided further insights into the genetic causes of various cardiomyopathies by creation of isogenic‐control lines (mutation correction in diseased hiPSCs or mutation creation in wild‐type hiPSCs)^105^, ^106^, ^107^ and by evaluating the potential role of variants of unknown significance and modifier genes.^108^

The hiPSC‐based modelling studies, however, also identified some limitations of this approach; namely the heterogeneous and relatively immature nature of the generated hiPSC‐CMs (consisting of different early‐stage cardiomyocyte subtypes), the use of single‐cells or non‐complex tissue models, and limitations associated with existing phenotyping methodologies. Recent work in the field has attempted to address the aforementioned limitations^109^ (*Figure* *5*). First, development‐biology inspired differentiation systems were established to coax the differentiation of hiPSC into purified populations of atrial, ventricular and conduction system (sinoatrial node) cells.^110^ Moreover, relevant non‐myocyte cardiac cells such as vascular, cardiac fibroblasts, epicardial and endocardial precursor cells could also be generated, allowing to construct more clinically relevant cardiac‐like structures and asses the role of non‐myocyte/cardiomyocyte crosstalk in cardiac pathologies. Similarly, ongoing efforts are focused on enhancing hiPSC‐CM maturation through the use of thyroid and glucocorticoid hormones,^111^ the effects of extracellular‐matrix and tissue‐engineering strategies, mechanical and electrical training, changes in the metabolic fuel to free fatty acids, and other interventions.

Moving from the cellular level to three‐dimensional engineered heart tissues, by combining polymer‐based scaffolds with hiPSC‐CMs, allowed to derive more clinically relevant models to study cardiomyopathies.^112^ The next frontier in tissue engineering will focus on establishing multi‐cellular cardiac‐like structures (for example through self‐organization in case of cardiac organoids^112^, ^113^) and on incorporating anatomical structural features through the use of organ decellularization/recellularization or three‐dimensional bio‐printing strategies^114^ (*Figure* *5*). Finally, innovative methodologies have been developed to characterize and modulate the functional properties of the hiPSC‐based cardiomyopathy models^115^, ^116^, ^117^ (*Figure* *5*). These diverse methodologies, focusing on characterizing the electrophysiological and mechanical properties at both the cellular^111^ and tissue levels,^106^, ^112^, ^117^ can be tailored to derive detailed pathophysiological mechanistic insights into various disease pathogenesis as well as scaled up for high throughput pharmacological studies.

### Specific therapies: genetic modification

The possibility of manipulating DNA through genome editing has enabled many advances in cardiovascular medicine, especially in terms of understanding genetic diseases but also of developing new targeted therapies. These techniques could quickly create opportunities to cure genetic cardiomyopathies associated with mutations in major determinants of the electrical or contractile properties of cardiomyocytes. The available techniques and the strategies to target cardiovascular cells have been extensively reviewed elsewhere.^118^, ^119^, ^120^, ^121^ Whereas the first genome editing experiments have been conducted in the late 1970s, CRISPR/Cas9 has recently emerged as the major technology used for therapeutic genome engineering, including for cardiomyopathies.

Genome editing in large animals appears a highly desirable model situation bridging basic proof of principle studies to clinical application. In this regard, pathologies where a number of mouse models have demonstrated applicability of a therapeutic AAV‐based CRISPR/Cas9 approach, stand out. A prominent example is Duchenne muscular dystrophy (DMD), where dystrophin gene mutations lead to absence of the dystrophin protein. Here, gene editing may suit the purpose of exon skipping, which results in a shortened, but functional protein. For example, several groups have demonstrated in a mouse model of DMD (mdx mouse) that excision of the mutated exon 23 suffices to enable expression of a stable dystrophin gene.^122^, ^123^, ^124^, ^125^ Functional assessment, though limited due to a mild phenotype of the mouse model, suggested improvement of the skeletal and heart muscles after local or systemic vector application. Extending this evidence, Amoasii et al.^126^ applied single‐guide RNAs and *Streptococcus pyogenes* Cas9 into DMD dogs (lacking exon 50), either intramuscularly (i.m.) or intravenously (i.v.). They found that dystrophin was expressed at the injected sites (i.m.) and also in the heart (i.v. approach). In a complementary transgenic pig model lacking exon 52 of the dystrophin allele, a well‐established Cas9 split version was used, where both halves were ligated to a pair of inteins (an intein is a protein sequence embedded within a precursor protein, and it can excise itself via protein splicing)^127^ which splice themselves out after recombination of the whole Cas9 RNA. In this setting, two guide RNAs (gRNAs), capable of excising exon 51 were evenly distributed on the same vectors. In pilot i.m. experiments, the intein‐split Cas9‐gRNA approach was efficient in editing up to 78% of the muscle nuclei analysed after regional application and up to 52% after systemic application of the AAV‐Cas9‐gE51 vectors. Moreover, in the heart, DMD expression was detected upon i.v. application 4 weeks postnatally. Up to 7% of the genomes were edited, sufficing to avoid fibrosis of the left ventricle. High‐resolution electrophysiological maps of the left ventricles indicated that the extent of low‐amplitude areas, corresponding to fibrotic myocardial tissue, was decreased after AAV9‐Cas9‐gE51 treatment. In accordance, treated DMD animals lived up to one third longer than untreated DMD animals, which displayed the phenotype of sudden cardiac death.^128^ In complementary experiments of hiPSC‐CMs lacking dystrophin exon 52, additional excision of exon 51 allowed re‐expression of myocytic genes, such as *MYH1*, *TTN* and cadherin 15 (*CDH15*). Only thereafter, DMD myoblasts were able to differentiate to multinucleated myocyte fibres, which were rarely found in untreated iPSC‐derived myoblast cultures under further differentiation protocols. Patient‐derived DMD iPSC‐CMs lacking exon 52 displayed an arrhythmogenic phenotype, which was normalized by AAV6‐Cas9‐gE51 treatment. Further refinements of the approach pending, AAV9‐Cas9 combinations might become applicable to DMD patients, offering exon excision to allow expression of a shortened but stable and functional dystrophin protein.

CRISPR/Cas9 is also a versatile tool that can be modified by deactivating the catalytic domains (forming a so‐called dead Cas9 or dCas9) and then fusing new functional models. Using dCas9, a new approach called ‘base editing’, has recently been reported as allowing direct replacement of a single base pair in a genome without the need for DNA cleavage (i.e., C‐G to T‐A conversion or a A‐T to G‐C transition).^129^, ^130^ The technique is still in its early developments but has recently been successfully applied in a murine model of Hutchinson–Gilford progeria syndrome caused by a mutation in the gene encoding nuclear lamin A.^131^ The base editing system packaged into an AAV9 was administered via a single retro‐orbital injection, and resulted in a durable correction of the pathogenic mutation (around 20%–60% across various organs 6 months after injection), reduction in the progerin protein levels and improved survival of animals. *In vivo* base editing thus represents another future therapeutic option for genetic cardiomyopathies.

### Novel modalities: drugs

Our increased understanding of genetics, epigenetics, proteomics, metabolomics and protein modulations associated with different morbidities have stimulated the development of innovative compounds able to successfully interfere with previously ‘un‐druggable’ targets. Especially genetics and the analysis of large‐scale next generation sequencing combined with phenotypic analysis turned out to be a very powerful tool in modern pharmaceutical research and drug target identification processes.^132^ This also led to a significant increase of the ‘druggable’ genome from early estimates of about 10%–14% of the number of proteins in the proteome in 2002 by Hopkins *et al*.,^133^ with an addition of 2282 genes to about 4479 (22%) of the 20 300 protein‐coding genes which are now deemed drugged or druggable.^134^ This number is likely to increase even further due to the inclusion of even more novel modalities, particularly the application of novel therapeutics based on nucleic acids. Nucleic acid‐based drugs, especially oligonucleotides, present unique opportunities to interfere with protein metabolism at the genome or gene level. Oligonucleotides can be categorized into four types:

- Single strand antisense oligonucleotides (ASOs) that cause knockdown via RNase H, RNA blockade through binding, splice switching or exon skipping.
- Double stranded small interfering RNAs (siRNAs) that feed into the RNA interference pathway.
- Single stranded anti‐miRs or microRNA precursors.
- mRNAs and CRISPR‐based therapies.

In general, oligonucleotides prevent the production of disease‐related genes by modulation of gene expression. Successful examples are the first ASO Vitravene (fomivirsen), approved in 1998, the first aptamer Macugen (pegaptanib), approved in 2004, and the first siRNA Onpattro (patisiran), approved in 2018 (*Table* *1*). New approaches also include the use of locked‐nucleic acid‐based technologies to block pathological cardiovascular microRNAs.^135^, ^136^ MicroRNA inhibitors or precursors may target altered gene expression patterns induced by a specific mutation or may intervene with pathophysiological secondary consequences of cardiomyopathies. Here, safety and efficiency of anti‐miRs have been documented in clinical studies.^137^, ^138^, ^139^ Moreover, a worldwide first study has shown safety and initial efficacy hints for miR‐132 blockade in chronic HF patients in a phase 1b study.^137^ A recent article reported on successful treatment of transthyretin amyloidosis using CRISPR‐Cas9 *in vivo* gene editing.^140^ And increased knowledge on epigenetic information in the setting of HF allows new diagnostic and therapeutic purposes in patients, as recently discussed.^141^

**Table 1:** All currently known nucleic acid‐based therapeutics in clinical research

Drug	Type	Mechanism	Application	Disease	Clinical trial identifiers (https://clinicaltrials.gov/ct2/home)
Vitravene®	PS DNA	RNase H1	Intraocular	CMV retinitis	NCT00002187
Kynamro®	PS 2′ MOE	RNase H1	Subcutaneous	Familial homozygous hypercholesterolemia	NCT01475825
Spinraza®	PS 2′ MOE	Splicing	Intrathecal	SMA	NCT04591678; NCT02462759
Tegsedi™	PS 2′ MOE	RNase H1 (loss of TTR protein)	Subcutaneous	TTR amyloidosis	NCT04306510
Exondys®	Morpholino	Splicing	Subcutaneous	Duchenne	NCT01540409; NCT02286947; NCT02420379; NCT03992430; NCT03985878
Vyondys 53	ASO	Exon 53 skipping	Intravenous	Duchenne	NCT04708314; NCT02500381
Onpattro®	siRNA (in lipid nano particles)	Ago2 (loss of TTR protein)	Intravenous	TTR amyloidosis	NCT01617967; NCT03862807; NCT03997383; NCT04201418; NCT02510261; NCT01961921; NCT02939820; NCT01961921; NCT05023889
Givlari	RNAi	Targeting ALAS‐1	Subcutaneous	Acute intermittent Porphyria, hereditary	NCT03906214; NCT02082860 Several more studies finished and ongoing
Macugen	Polynucleotide	Aptamer (degrades VEGF)	Intravitreal	Age‐related macular degeneration; choroidal neovascularization	NCT00549055; NCT00320775 Several more studies finished and ongoing
Viltepso	ASO	Exon 53 skipping	Intravenous	Duchenne	NCT02740972; NCT02310906; NCT04337112
Defitelio	Mixture of single‐stranded oligonucleotides	Anti‐thrombotic, pro‐fibrinolytic	Intravenous	Severe hepatic veno‐occlusive disease	NCT00628498; NCT00358501

ASO, antisense oligonucleotide; CMV, cytomegalovirus; RNAi, RNA interference; siRNA, small interfering RNA; SMA, spinal muscular atrophy; TTR, transthyretin; VEGF, vascular endothelial growth factor.

So, we have significantly increased the druggable genome. Despite this, we need to overcome additional hurdles such as new challenges posed by cell, tissue and organ‐specific targeting. Additionally, it will be important to see to which extent financiers are willing to fund such personal medicine approaches in the cardiomyopathies in the future. Apart from that, cross functional and integrative approaches are needed, as can be seen in the recent successes of sodium–glucose co‐transporter 2 inhibitors (gliflozins) in different disease areas such as HF, diabetes and kidney disease.^142^ However, the increased focus of ‘big pharma’ in targeting rare diseases, the introduction of novel modalities combined with innovative experimental strategies such as organs on a chip and iPSC biology to exploit these opportunities, give rise to further optimism.

### Development of a targeted therapy for hypertrophic cardiomyopathy

Understanding the fundamental biophysical and biochemical changes that result from pathogenic HCM variants prompted a screen of small molecules to identify lead compounds that could modulate myosin ATPase activity. One compound, MYK‐461 was found that reduced ATPase activity and myofibril contractility in a dose‐dependent fashion.^143^ Subsequent analyses demonstrated that MYK‐461 normalized DRX and SRX ratios in heart tissues and iPSC‐CMs with pathogenic HCM variants, reduced hyper‐contractility and normalized relaxation and improved energetics.^90^, ^144^ Moreover, young HCM mice treated with MYK‐461^143^ prevented clinical disease during treatment duration. MYK‐461 was developed as mavacamten, a first‐in‐kind direct oral treatment for HCM. Pre‐clinical and early clinical trials in human patients demonstrated the safety and improved ventricular compliance in treated HCM patients.^145^, ^146^, ^147^ A recent phase III, double‐blind, randomized, placebo‐controlled trial (EXPLORER‐HCM) of patients with obstructive HCM (gradient ≥50 mmHg) showed that treatment with mavacamten for 30 weeks improved exercise capacity, New York Heart Association class, symptoms, and reduced outflow tract obstructive gradients without significant changes in LVEF.^12^ Preliminary analyses of cardiac magnetic resonance imaging in a small subset of EXPLORER‐HCM participants showed notable improvements in cardiac morphology, including significant reductions in LV mass and left atrial volumes. Together these data indicate the considerable potential for directly targeting the sarcomere to alleviate altered biophysical abnormalities and adverse ventricular remodelling in HCM, and raises the prospect that treatment will reduce the risk for AF and progression to HF in HCM patients.

Considerable need for further mechanistic studies of HCM remains. Experimental studies of mavacamten demonstrate rescue of abnormal myofilament calcium sensitivity that occurs with pathogenic variants in thin filament sarcomere proteins.^148^ However, these salutary effects were accompanied by partial or sometimes worsening effects on contractility, through uncertain mechanisms. The signals and molecules that evoke distinct patterns of hypertrophy – including strikingly asymmetric septal involvement or apical remodelling – and how these influence mitral valve morphology and function remain an enigma. Once again, human genotypes may help to illuminate these processes. With the recent identification of many polymorphic variants across the genome that may influence disease expression, many new opportunities to further explore disease pathogenesis that may advance additional new therapeutics for HCM will arise.

In 2003, Crilley and colleagues showed that HCM caused by sarcomere gene mutations is characterized by impaired energy metabolism evident from a reduction in *in vivo* phosphocreatine to ATP (PCr/ATP) ratio measured by magnetic resonance spectroscopy.^149^ The PCr/ATP ratio is an indirect measure of energy status, and correlates with severity of HF. Notably, the reduction in PCr/ATP was observed in individuals without cardiac hypertrophy, suggesting that impaired energetics is a primary defect in the onset of HCM. Subsequent studies, which combined measurements of oxygen consumption with acetate by positron emission tomography and cardiac work by cardiac magnetic resonance imaging, showed reduced myocardial efficiency in both pre‐clinical sarcomere mutation carriers and patients with (obstructive) HCM.^150^, ^151^ This reduction in cardiac efficiency was present in individuals with mutations in thick filament genes *MYH7* and *MYBPC3* ^150^, ^151^ and the thin filament gene *TNNT2*,^152^ and at the pre‐clinical stage was explained by increased oxygen consumption. Multiple cellular pathomechanisms may underlie increased oxygen consumption and reduced cardiac efficiency, which already manifests at the very early disease stage before cardiac remodelling. Increased myofilament Ca^2+^ sensitivity, increased kinetics and ATPase activity, and disordered myosin have been proposed to underlie impaired energy metabolism.^89^, ^91^

Therapies targeting cellular mechanisms that underlie reduced cardiac efficiency, or increase energetics via modulating metabolism and mitochondrial function, may improve cardiac function and prevent cardiac remodelling.^147^, ^153^, ^154^ Clinical trials mostly focus on patients with manifested symptomatic HCM, and reported benefit of therapies targeting myosin and/or metabolism. Future studies are warranted in pre‐clinical mutation carriers, using clearly defined endpoints, to establish if treatments aimed to improve cardiac energetics prevent and/or reverse cardiac dysfunction and remodelling.^155^

## Future directions – on the horizon

Currently, more than 60 million people are suffering from HF worldwide, and numbers are expected to increase in the future. One third of them have non‐ischaemic cardiomyopathies. In contrast to ischaemic and valvular heart disease, where new coronary and valvular interventions have exploded and revolutionized its treatment, we have no targeted therapies for DCM or HCM. In order to get precision medicine, we need to distinguish the different phenotypes. Especially, patients with genetic cardiomyopathies, including LMNA, RBM20, TTN subgroups or PLN, but also DCM related to chemotherapy or pregnancy are poorly responsive to classical HF therapies. And in HCM, it has become clear that direct toxic effects of aberrant protein play a role in the onset and progression of disease. Combining functional and molecular phenotyping in human cardiac samples for target discovery followed by experimental validation, along with machine learning‐guided ECG and imaging analyses, will allow not only identification of novel therapeutic targets for precision medicine in individual genetic or acquired cardiomyopathies, but also validate and repurpose existing drugs.

### Funding

Prof. de Boer is supported by grants from the Dutch Heart Foundation (CVON SHE‐PREDICTS‐HF, grant 2017‐21; CVON RED‐CVD, grant 2017‐11; CVON PREDICT2, grant 2018‐30; and DCVA DOUBLE‐DOSE, grant 2020B005), by a grant from the Leducq Foundation (Cure PhosphoLambaN‐induced cardiomyopathy; Cure‐PLaN), and by a grant from the European Research Council (ERC CoG 818715, SECRETE‐HF). Prof. Hulot is supported by grants from the French National Research Agency (NADHeart ANR‐17‐CE17‐0015‐02; PACIFIC ANR‐18‐CE14‐0032‐01; and CORRECT_LMNA ANR‐19‐CE17‐0013‐02), Fédération Française de Cardiologie, the Fondation pour la Recherche Médicale (EQU201903007852), a University Research Federation against heart failure (FHU2019, PREVENT_Heart Failure), and by a grant from the Leducq Foundation (18CVD05). Dr. Knöll is supported by grants from AstraZeneca, Hjärt och Lungfonden, and Deutsche Forschungsgemeinschaft (DFG). Prof. Kupatt is supported by the German Research Foundation (DFG SFB‐TRR127‐A2, SFB‐TRR 267‐B8) and the German Center for Cardiovascular Research (DZHK). Prof. Linke is funded by grants from the German Research Foundation (SFB1002‐TPA08) and the IZKF Muenster (Li1/029/20). Prof. van der Velden acknowledges support from NWO‐ZonMW (91818602 VICI grant), ZonMW and Heart Foundation for the translational research program, project 95105003; the Dutch Cardiovascular Alliance (DCVA) grant Double Dose 2021; and the Leducq Foundation grant number 20CVD01. Prof. Thum is supported by the EU (Grant Cardioregenix, GA 825670), the Deutsche Forschungsgemeinschaft DFG (TH903/20‐2 and INST 95/15641).

**Conflict of interest:** The UMCG, which employs Prof. de Boer, has received research grants and/or fees from AstraZeneca, Abbott, Boehringer Ingelheim, Cardior Pharmaceuticals GmbH, Ionis Pharmaceuticals, Inc., Novo Nordisk, and Roche. R.A.d.B. received speaker fees from Abbott, AstraZeneca, Bayer, Novartis, and Roche. S.H. received personal fees for scientific advice from AstraZeneca, CSL Behring, Cellprothera, Bayer and Merck; and an unrestricted research grant from Pfizer. A.J.S.C. declares no conflicts related to this work. Outside of this work, in the last 3 years, he declares having received honoraria and/or lecture fees from AstraZeneca, Bayer, Boehringer Ingelheim, Menarini, Novartis, Nutricia, Servier, Vifor, Abbott, Actimed, Arena, Cardiac Dimensions, Corvia, CVRx, Enopace, ESN Cleer, Faraday, Gore, Impulse Dynamics and Respicardia. S.D. holds patents on microRNA therapeutics. T.E. and L.C. hold a patent on gene‐therapy vectors for treating cardiomyopathy that was licensed to DiNAQOR AG, are members of the DiNAQOR Scientific Advisory Board, and have shares in DiNAQOR. The APHP, which employs Prof. Hulot, has received research grants from Bioserenity, Sanofi, Servier and Novo Nordisk. Outside of this work, J.S.H. has received speaker, advisory board or consultancy fees from Amgen, AstraZeneca, Bayer, Bioserenity, Boehringer Ingelheim, Bristol‐Myers Squibb, MSD, Novartis and Novo Nordisk. R.K. is an employee of AstraZeneca. C.K. holds a patent on AAV‐based gene editing for Duchenne muscular dystrophy and has received advisor or speaker fees from AstraZeneca and AskBio. C.E.S. is a founder of Myokardia (a Bristol‐Myers‐Squibb Subsidiary); a consultant of Maze and BridgeBio; and is member of the Board of Directors at Merck. C.G.T. has received funding from Amgen and personal fees from Vivalyfe, outside of the submitted work, and is listed as an inventor on two heart failure patents. Prof. Seferovic declares consulting fees for Boehringer Ingelheim, Novartis, Vifor Pharma; and honoraria for lectures for Servier, AstraZeneca, Respicardia, Boehringer Ingelheim, and Novartis. T.T. has filed and licensed patents in the field of noncoding RNAs; he is founder and shareholder of Cardior Pharmaceuticals GmbH and is on the advisory board and/or received speaker fees from Novo Nordisk, Boehringer Ingelheim, Takeda, Amicus Therapeutics, Ksilink and Sanofi‐Genzyme.
